# Antibiotic governance and use on commercial and smallholder farms in eastern China

**DOI:** 10.3389/fvets.2023.1128707

**Published:** 2023-03-17

**Authors:** Binjuan Liu, Wei Wang, Ziru Deng, Cong Ma, Na Wang, Chaowei Fu, Helen Lambert, Fei Yan

**Affiliations:** ^1^Department of Population Health Sciences, Bristol Medical School, University of Bristol, Bristol, United Kingdom; ^2^Key Laboratory of Public Health Safety, NHC Key Laboratory of Health Technology Assessment, School of Public Health, Fudan University, Shanghai, China; ^3^Division of Health Economics, Policy and Management, School of Public Health, The University of Hong Kong, Hong Kong, China

**Keywords:** antibiotics, governance, agriculture, smallholder, commercial farm, China, antimicrobial resistance, one health

## Abstract

**Introduction:**

China is one of the largest consumers of agricultural antibiotics in the world. While the Chinese government has been tightening its regulations to control antimicrobial resistance (AMR) from animal sources in recent years, the extent of antimicrobial oversight and the practices of antibiotic use in animal agriculture in China has not yet been explored. This study describes the practices of antimicrobial management in eastern China and current scenarios of antibiotic use in commercial farms and smallholder backyard farming.

**Methods:**

33 semi-structured interviews were conducted with government agriculture officials, veterinary drug sellers, farmers and smallholders in two contrasting areas of rural Zhejiang and Jiangsu provinces, China. Interview transcripts were analyzed in NVivo12 using a thematic approach.

**Results:**

Findings revealed that although the governance of antibiotic use has made progress, especially in controlling irrational antibiotic use in commercial farms, smallholders are under-regulated due to a lack of resources and assumptions about their marginal role as food safety governance targets. We also found that smallholders resort to human antibiotics for the treatment of backyard animals because of economic constraints and lack of access to professional veterinary services.

**Discussion:**

More attention needs to be devoted to the local structural needs of farmers to reduce antibiotic misuse. Considering the extensive links of AMR exposure under the One Health framework, efforts to integrate smallholders in antibiotic governance are required to address the AMR burden systematically in China.

## 1. Introduction

AMR is one of the most serious global public health threats. Although resistance to antimicrobials is a natural phenomenon, the widespread use of antibiotics has been creating selective pressure and accelerating the screening of drug-resistant strains (1). Globally, animal agriculture accounts for over half of all antibiotic consumption (2). Antimicrobials are commonly used in livestock farming to prevent infections and as growth promoting agents (3), which can aggravate the problem of AMR selection. Global antimicrobial use in the agriculture sector is expected to increase due to accelerating demand for animal-source nutrition, especially in low- and middle-income countries (LMICs) (4).

AMR has been recognized as a One Health issue due to increasing evidence of the development and transmission of AMR through human-animal-environmental interactions (5, 6). Misuse and overuse of antibiotics in animal agriculture can increase the risk of human exposure to AMR through the food chain and environmental pathways (7, 8). Food animals and the contaminated environments in agricultural settings can serve as reservoirs of antibiotic-resistant bacteria (ARB) and antibiotic-resistant genes (ARGs) (9). The chance of human exposure to resistant pathogens will increase through direct and indirect contact with contaminated food animals, animal products, water, soil, sludge, and manure (10). Hence, optimizing antibiotic use in animal agriculture is critical for tackling AMR from a One Health perspective (6).

China is one of the leading consumers of global agricultural antibiotics. A study estimated that China consumes approximately 162,000 tons of antibiotics in 2013, of which 52% were used in agriculture (11). It is estimated that the share of global antibiotics consumption in food animals for China will increase from 23% in 2010 to 30% in 2030 (2). Surging antibiotic use in agriculture and the associated antibiotic residue contamination of food and the environment are contributing to severe bacterial resistance in China (12–14), as well as in Europe and worldwide (15, 16). In the past two decades, the Chinese government has announced a series of regulations to control agricultural antibiotic use, including releasing a prohibited list of veterinary antibiotics, strengthening management of withdrawal period and medicine record, classified management of prescription and non-prescription veterinary drugs, and publishing national action plans to regulate the veterinary use of antimicrobials (17–20). As a result of increased regulation, the consumption of antibiotics in China's agricultural sector had fallen from 69,292 tons in 2014 to 30,903 tons in 2019, according to official veterinary bulletin published by Ministry of Agriculture and Rural Affairs (21). Despite this, studies found that misuse of antibiotics in animal agriculture is still prevalent in China: a survey of farmer's antibiotic use on small and medium chicken farms in Ningxia, China found that three-quarters of respondents misused antibiotics and still used antibiotics on the government prohibited list (22); another survey of large-scale and smallholder pig farmers in a county in Yunnan Province found more than 90% of respondents reported they can purchase antibiotics without prescription (23). There is a dearth of evidence on the practices of policy enforcement and drivers of misuse of veterinary antibiotics.

This study was conducted to understand the practices of enforcement of antimicrobial regulation and antibiotic use in animal agriculture in China. We interviewed 33 local stakeholders, including government agriculture officials, veterinary drug sellers, commercial farmers and smallholders, in two counties in eastern China to obtain local accounts of antimicrobial regulation enforcement and antibiotic use in commercial and smallholder backyard farming.

## 2. Materials and methods

### 2.1. Background: Antibiotic policy and governance in China's animal agricultural sector

This study was conducted within the context of China's efforts to tighten its regulations on antibiotic use in the agricultural industry. In 2016, triggered by the discovery of MCR-1 in pigs in 2015 (24), China announced a ban on the use of colistin for growth promotion in livestock. In recent years, China has launched a series of regulations to reduce veterinary antimicrobial use following the National Action Plan to Combat Antimicrobial Resistance from Animal Resources (2017–2020) (18). Since 2018, the Ministry of Agriculture and Rural Affairs (MARA, *nongye nongcun bu*) has designated 100 pilot livestock farms each year to trial the Veterinary Antimicrobial Use Reduction Action (experiences from the trial have fed into formulating the National Action Plan for Veterinary Antimicrobial Use Reduction (2021–2025) (20)). MARA launched a regulation in 2019 to withdraw medicated feed additives, stipulating that antimicrobials can be used for veterinary medicine but not for veterinary medicine additive purposes (25), also known as the “Feed Antimicrobial/Antibiotic Ban”(*siliao jin kang ling*). According to the announcement, all growth-promoting feed medications are banned except for traditional Chinese medicine (26).

Since the introduction of Veterinary Antimicrobial Use Reduction Action and the feed antimicrobial ban, a variety of provinces and cities across China have started top-down antibiotic reduction and “antibiotic-free” initiatives. “Antibiotic-free farming” has been promoted by several provincial or municipal governments for lowering dependence on antibiotics in animal agriculture. These initiatives include building pilot antibiotic-free farming sites, training farmers in antibiotic-free farming techniques, and setting up industry associations that integrate farms, research institutes, testing institutes, slaughtering enterprises, and supermarkets to promote antibiotic-free animal products (27, 28).

#### 2.1.1. Oversight of veterinary drug use in animal meat production

**Figure 1:**
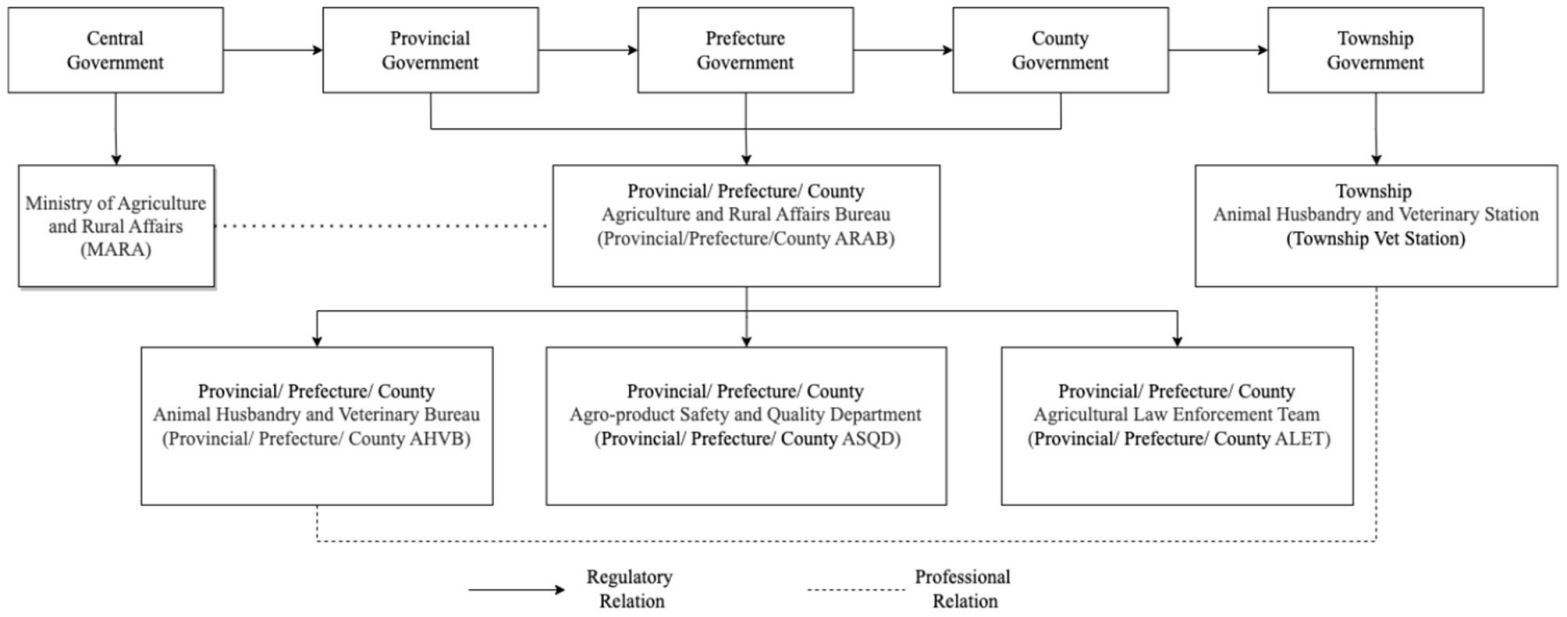
Governance system of veterinary drug use in animal meat production in China.

In China, the responsibility of antimicrobial use oversight is shared among MARA, the Ministry of Health (MoH), and the China Food and Drug Administration (CFDA). While MoH regulates antimicrobial use in human health sector and CFDA regulates the registration, production, distribution and quality control of antimicrobials, MARA exclusively manages antimicrobial use in animal meat production. MARA, formerly the Ministry of Agriculture (MOA), which is in charge of works related to farmers, agricultural activities, and rural areas, is responsible for veterinary drug use oversight in animal meat production at the national level. Each provincial, prefecture, and county-level government has a corresponding Agriculture and Rural Affairs Bureau (ARAB, *nongye nongcun ju*), and all provincial, prefecture, and county-level ARABs are involved in veterinary drug use oversight in animal meat production within their jurisdiction (Figure 1). The food safety supervision system additionally regulates veterinary drug residues in agricultural products. In the food safety supervision system, ARABs are responsible for the oversight of agricultural products during planting, breeding, and slaughtering. The Market Supervision and Regulation Bureaus (MSRBs, *shichang jiandu guanli ju*) are responsible for supervision of agricultural products during the distribution stage. The Food Safety Committees (FSCs, *shipin anquan weiyuanhui*) are responsible for coordination of food safety supervision across different departments.

In each provincial, prefecture and county ARAB, veterinary drug use oversight is carried out by sub-departments of Animal Husbandry and Veterinary Bureau (AHVB, *xumu shouyi ju*), Agro-product Safety and Quality Department (ASQD, *nongchanpin zhiliang anquan jianguan ke*), and Agricultural Law Enforcement Team (ALET, *nongye zhifa dadui*) (Figure 1). The responsibilities of ASQDs include agricultural product quality and safety monitoring, traceability, and risk assessment. ASQDs are responsible for carrying out random inspections primarily on agricultural production enterprises, large farms, demonstration sites, specialized cooperative economic organizations and slaughterhouses. The main detection indicators include prohibited pesticides, drugs, and other prohibited chemical compounds, and this includes testing for the presence of antimicrobials (see Supplementary Table 1) (29). ALETs in turn are responsible for investigating and dealing with illegal practices. AHVBs are responsible for matters relating to animal husbandry and slaughtering, veterinary services, drugs, feed and additives, and animal epidemic prevention and quarantine. Training and guidance on agricultural production, drug use, quality and safety management for farmers are also provided by AHVBs.

The Township Animal Husbandry and Veterinary Station (*xiangzhen xumu shouyi zhan*, “Vet Station (*shouyi zhan*)” for short) is the grassroots level veterinary public service institution under supervision of both the county-level AHVB and the township government. The vet station usually incorporates several Official Veterinarians (*guanfang shouyi*, OVs), defined by the Chinese Official Veterinarian System as law enforcement officers authorized by the government, who are engaged in health supervision work of animals and animal products and have the right to issue animal health certificates (30). Vet stations make direct contact with farmers and are responsible for drug residue inspections, disease prevention, policy dissemination and agricultural technical extension services. Vet stations also facilitate management work related to animal husbandry and veterinary service that carried out by administrations above the township level.

### 2.2. Methods and setting

#### 2.2.1. Study setting and participants

The study was conducted in D county, Zhejiang Province and H district (in China a district is an integral part of a city, whose administrative level is equivalent to a county), Jiangsu Province, both locate in the Yangtze Delta region, eastern China, which has a subtropical monsoon climate with well-marked four seasons and plenty of rainfall and sunlight. Mixed crop, poultry, and aquaculture farming in smallholder households or small to medium-sized farms is predominant in both locations.

To understand the current status of antibiotic governance and practices of antibiotic use in animal agriculture in China, we conducted semi-structured interviews with a total of 33 stakeholders in the agriculture sector in eastern China from July 2020 to January 2021. The aim was to obtain a range of views from those involved in antibiotic governance and use at all levels locally. Our interviewees included six agricultural officials across various departments, five feed and drug store owners, and twenty-two farmers from two counties (for informant characteristics see Table 1). Both purposeful sampling and snowballing were used to recruit participants.

**Table 1:** Informant characteristics.

**Stakeholders**		**Age range**	**Education**	**Number**
	County AHVB official			2
	County ASQD official			2
Agricultural Official	Township ARAB official	36–58	Junior college to postgraduate	1
	Township VB official			1
Veterinary feed and drug store owner^a^		35–65	Primary school to junior college	5
Farmer	Commercial farmer^b^	45–75	Primary school to secondary school	13
	Smallholder^c^			9

^a^Feed and drug store owners operate on a small scale and customers are mainly local smallholders.

^b^Commercial farmers interviewed run small or medium sized aquaculture (mainly freshwater shrimp and fish) or livestock farming as a family business in D county. Their animals and animal products were sold to restaurants and supermarkets in the nearby townships and cities.

^c^Smallholders interviewed engage with backyard livestock (mainly poultry) farming in the H district, their animals are usually less than 50 and are consumed within households.

#### 2.2.2. Data collection

Initial contact with key informants in the villages was established through contacts from the local Center for Disease Control (CDC). Field entrance and introduction to the participants were facilitated by key informants.

Farmers' interviews were conducted on-farm, and interviews with agricultural officials and feed and drug store owners were conducted in their workplaces. Interviews with agricultural officials focused on the institutional responsibilities of antimicrobial oversight, regulatory status and existing problems, while those with veterinary feed and drug owners focused on drug sales and their perceptions of antimicrobial regulation. Interviews with farmers and smallholders focused on breeding and antibiotic use practices and their understandings of antimicrobial regulation. Interviews and transcription of recordings were carried out in mandarin.

#### 2.2.3. Data analysis

Interview transcripts were analyzed within the qualitative data analysis software Nvivo12. Thematic data analysis technique was used to identify salient themes from across the data sources. Initial codes and sub-codes were developed independently by team members using a small number of transcripts covering different types of stakeholders. A bilingual codebook was developed by all team members after cross-review and discussion. In addition to high level codes and sub-codes applicable to all types of stakeholders (e.g., “perceptions of antibiotic governance”), we also developed sub-codes specific to each type of stakeholder (e.g., “biosecurity measures” for interviews with farmers, “interdepartmental cooperation” for interviews with agricultural officials). Each transcript was coded by two individual researchers. Recurrent codes and themes were identified by iterative reading and coding. Data was organized and analyzed by theme. The results of data analysis are presented below.

## 3. Results

The results are presented in two parts. The first section below reports findings on the implementation of antimicrobial governance, drawing mainly from interviews with agricultural officials, including how random inspection and policy dissemination is carried out at the local level and the obstacles and concerns that officials have in their everyday management work. The subsequent section describes different scenarios of antibiotic use practices in commercial and smallholder farming.

### 3.1. Implementation of antimicrobial oversight

#### 3.1.1. Policy dissemination and training

Officials identified policy dissemination and training as an important part of their administrative work. County administrations contact township administrations to issue handbooks, leaflets, and commitment letters to farmers. The county administrative authorities also try to convene the staff from large-scale farms for policy dissemination and training at county headquarters two to four times a year. However, officials reported that due to time and transportation inconveniences, the participation rate in these meetings is not high.

“*Most large-scale farmers are busy, not only large-scale farms, that is to say, they are all busy. For example, some farmers are going to deliver eggs in the morning, and it is difficult to ask them to have a meeting to spare time…… some are elderly ones [who] raise animals in their own household. Like these individuals, if [we] ask them to come to meetings, there is no transportation at all. To be honest, we can only distribute leaflets through township agricultural offices. We just choose a few bigger ones to go to, or select a few villages.”* (Interview 08, Agricultural official from D county)

The county agricultural official said that even though small farms are hard to manage and sometimes there are gaps in terms of dissemination, in their view this is not a serious problem. To the officials, the lack of engagement with smallholders is justified by the fact that animals raised on smallholdings are small in number and mainly for domestic consumption.

“*Some are elderly people, they don't raise much, they may be illiterate. Actually, they don't know how much to use based on the drug packaging. They are all based on their experience, right? Maybe they exceed the standard amount. But basically, they are eaten by themselves, not sold in the supermarket. It's not a big problem. What we can do is inform them, there may be cases that we miss [of] some individual farmers, we can only do our own responsibility.”* (Interview 08, Agricultural official from D county)

Township ARA offices or Vet Stations provide training to farmers through farmer meetings in the village or face-to-face consulting. Township agricultural officials from D county reported that if farmers do not come to the village meeting, it is their responsibility to meet the farmers face-to-face to ensure that dissemination reaches all households. One official expressed concern that the training has little influence on farmers' drug use because they would not understand pharmaceutical information fully.

“*We should publicize every household, send out some notices and lists of prohibited drugs banned by the state, and let them know for themselves. In this process, the most difficult point is that farmers have low education. You show this to him, he can't read. You explain this to him, and he says I know. When he went to the store to buy medicine, he only heard that when shrimps are sick seemed to need some medicine. He doesn't know what the medicine is like, and he couldn't see what was going on.”* (Interview 10, township agricultural official from D county)

The commercial farmers in D county receive regular training from township ARA offices, and most of them remember the drugs on the prohibited list. However, one farmer reported that the training is too “theoretical” and wanted more practical information for guiding food production:

“*What we want to hear is something more concrete, like how long the culture time for the shrimp is, how to adjust the water quality, what drugs to use, how to prevent diseases, and so on. Rather than listening to nitrite, ammonia nitrogen, and things like this.”* (Interview 07, shrimp farmer from D county)

Smallholders in H district did not mention training or reported they don't receive any training or education and questioned the need for this:

“*No one has come to give any information. We have so few[chickens], we're not like large scale farms.”(Interview 25, smallholder from H district)*

“*It doesn't make a difference if they don't [come]. In any case, we know what to do ourselves……We're too old now to go and participate in that kind of activity.”(Interview 20, smallholder from H district)*

#### 3.1.2. Drug residue detection

Drug residue detection is one of the primary means through which the county/district ARAB exercised oversight of veterinary drug use. Antimicrobial residue testing is incorporated into the agro-product safety and quality oversight led by the county/district ASQD (for testing indicators see Supplementary Table 1). AHVB and ALET are in charge of the sampling of farmed animals. Tests are done by third-party companies that have contracts with the government, using testing indicators in accordance with government guidelines. The ASQD at each administrative level monitors and tests food animals in its jurisdiction, and the testing regime has annual targets for the number of tests, coverage level, and compliance rate. The target farms for testing are chosen in rotation within the county or randomly selected from the government databases of registered farms. Information about the testing entities and quantities is registered on databases such as the National Agricultural Food Quality and Safety Traceability Management Information Platform (http://qsst.moa.gov.cn/) or provincial platforms.

One problem with selecting farms from the information platforms is that the registered entities are mostly large-scale farms, which means that only large-scale farms will be selected for random testing by the upper-level authorities like MARA and provincial ARABs. As the agricultural official quoted below explains, the unstable conditions of small farms make it more difficult to keep records of their farming information and monitor them.

“*Some people say were maybe rearing fish last year in a pool, but that didn't earn them any money, so this year they have switched to something else. When that's no good again they change to something else. So the fluctuations are pretty big. Yes, so in this way, it is in fact all quite difficult to keep monitoring.”* (Interview 09, Agricultural official from D county)

According to an ARAB official from D county, each township ARA office is required to set up a testing unit for township-level agro-product safety and quality oversight testing and the public to test their agricultural products (consumers can bring products they have purchased to test for pesticides and other food safety indicators). However, one township agricultural official reported that most of the township-level testing units do not test for antimicrobial residues but only heavy metal and pesticides, because of a lack of technical resources.

“*I think at the most grassroots level like us, if you want to monitor or test[antimicrobial residues], especially the aquatic products, the most effective and feasible method would be rapid testing, ……, I estimate that the equipment is very expensive, and requires technology we don't have. We do a test on this black fish, kill it, slice it, and make a sample and then half a day passed. Half a day, we still do not test many indicators. For antimicrobials, we don't have the technology and equipment.”* (Interview 10, township agricultural official from D county)

Almost all the commercial farmers interviewed in D county mentioned residue testing from the township or county once or twice a year, mostly at the time when the animals or animal/aquatic products are about to be sold. If their products pass the tests, then a product certificate is issued to the farmer. Interviews with smallholders in H district reflect the fact that these smallholders are rarely identified as targets for random testing.

#### 3.1.3. Food safety concerns: System linkages with market regulation

Agricultural officials made it clear that they are in charge of oversight of food safety during the animal production stage, whereas once the animal/animal product enters the market, oversight becomes the duty of the Market Supervision and Regulation Bureau. Problems found at the distribution or consumption stage would be reported to the Agricultural administrations for further investigation at the production stage.

Although there is no direct testing by the Market Supervision and Regulation team on commercial farms in D county, farmers are aware that their animal products will be tested once they enter the market. If residues of a drug on the prohibited list were found in the animal products, farmers could not only be fined but may also lose their regular customers or even the permits to breed livestock. This becomes an incentive for them to follow recommended drug administration practices such as not using drugs from the prohibited list and observing drug withdrawal periods.

“*If they are given any drugs within the previous two or three months and then the time comes to sell them, they take them to the market and if the testing department can detect that you've used antibiotics (kang sheng su) or prohibited drugs - they can detect it - then you get fined. Then after that, they wouldn't want the farmer's produce anymore and the farmer wouldn't be able to sell it off.”* (Interview 04, commercial farmer from D county)

Some farmers also describe the potential for oversight mechanisms such as random testing and provision of certificates to be “reassuring”, both for customers' food safety concerns and for themselves in securing a reliable customer base.

“*Before they are sold the authorities need to take blood samples and first test the samples before they can be sold. They do the examinations to see if they are over the indicated limits. You have a few dealers who are fixed, and they have to have samples taken. After our chickens have been reared to maturity, they all go to places like hotels and restaurants. They are regular clients, and all are outside of the region in high-end restaurants in HZ city. They also need to take samples. If whoever buys them tests them, then this would be more reassuring.”* (Interview 01, commercial farmer from D county)

On the other hand, when asked about antimicrobial regulation, the smallholders expressed greater concern about antibiotic use in large-scale commercial farms. Similar to the thinking of the agricultural official quote above (Interview 08 in 3.1.1 Policy dissemination and training), one farmer said that smallholders should not be the main oversight targets.

“*In my own mind this is what I think, but I can't generalize. My perspective is that those who are farming eggs, like big markets- I reckon that they all are using lots of antibiotics routinely……If they don't use them then the rate of egg production will not be high at all. What is happening with the smallholders with a few animals is not the point, it's those big farming operations.”* (Interview 19, smallholder from H district)

An agricultural official expressed concern for the potential gaps in food safety regulation such as lack of oversight of farmers' markets where individual farmers sell products on a stall directly to customers, and that the requirements for different markets are not standardized. One official gave the example that the government has been issuing quality and safety certificates for animal products and trying to make this a standardized process in the food supply chain. However, the agricultural administration ran into difficulties as different markets have their own standards and do not necessarily recognize or require such certificates:

“*There is perhaps something of a weak link in the matter of linking up with the markets, including many of those promotional initiatives. So it's the same with the promotion of those agricultural product certificates. There isn't a great deal of enthusiastic uptake on the part of the business entities. It doesn't make much of a difference whether or not you draw up the certificates for them. Then the unlicensed small retailers want these things even less, for example, if someone has set out their stall in the street, and the masses are coming to buy from their stall, they definitely aren't going to require that you display this kind of certification.”* (Interview 09, Agriculture official from D county)

### 3.2. Practices of antibiotic use on farms

#### 3.2.1. Transition from antimicrobials use to disease prevention on commercial farms

Farmers in D county mentioned how their antibiotic use practices have changed due to stricter regulations recently, especially in the last one or two years. Previously they used to be dependent on antibiotics, but now they tend to use progressively fewer antibiotics for their animals as regulatory oversight has increased. Farmers frequently mentioned awareness of the consequences if the testing resulted in prohibited drugs being identified; they might be fined or even deprived of the right to run a commercial farm. Water, sludge, and animal samples were tested regularly, several (2–5) times a year. Farmers also remembered how they were instructed during training not to use prohibited drugs and to use fewer antibiotics.

Following the Antimicrobial Reduction Action, farmers are recommended to use Chinese herbal medicine or other alternatives to antibiotics. When asked about disease management strategies, most of the commercial aquaculture farmers in D county highlighted the importance of maintaining water quality for disease prevention.

“*The government publishes information about everything, and they don't allow those types of antibiotic (kang sheng su) drugs to be used without rhyme or reason. With food products, we sometimes also go to training conferences, where the professors will explain everything. They don't allow you to use those kinds of antibiotic drugs just as you like, or other drugs that have been previously prohibited. Generally, we always use Chinese herbal medicines to treat and prevent illnesses, and some water quality disinfectants, changing the water, or something to kill the germs.”* (Interview 04, commercial farmer from D county)

Aquaculture farmers reported using only small amounts of enrofloxacin for prevention, following production cycles. Farmers in D county also reported no longer being able to buy feed containing antibiotics at feed stores since the Feed Antimicrobial Ban came into force. However, some loopholes remain in feed purchasing. For instance, a shrimp farmer said he could still ask the veterinary feed and drug store owners to add antibiotics into the feed at the time of purchase.

“*Q: Do you also give anti-inflammatory drugs for prevention?*

*A: Shrimp? It should be enrofloxacin and oxytetracycline*.

*Q: Is it written on the feed bag? What drugs were used?*

*A: No, tell them that you need to add some medicine, and they will add some for you. They made it for you.”* (Interview 07, commercial farmers from D county)

During our researcher's visit to a veterinary drug and feed store, we found prescription drugs for sale in the store and the owner was not aware that those drugs should only be sold with a prescription, suggesting that the farmer's account of obtaining antibiotics with relative ease from agricultural stores may not be exceptional.

Some farmers and agricultural officials identified “antibiotic-free” farming as a new trend as they heard about top-down initiatives and propaganda. In D county, we found one “antibiotic-free” farming product led by Farmers Cooperation Economic Organizations and a rabbit farmer who provided his views of what it takes to shift to antibiotic-free feed. We present these two cases to show how an antibiotic-free initiatives trial was conducted in D county.

Case 1: Antibiotic-free farming led by cooperation economic organization

YY town has around 13 square kilometers of aquaculture and farmers raise snake-headed fish, soft-shelled turtle, and freshwater shrimp. The town is known for its snake-headed fish with the brand name called “Black beauty (*heiliqiao*)”, which sells mostly to restaurants in Shanghai, Hangzhou and other places across the Yangtze river delta region. In recent years, Zhejiang has been establishing the Associations of Farmers Cooperation Economic Organizations (refers to “the Association” below), the self-operated non-profit social organizations funded by the government that unite the Farmers Cooperation Economic Organization (FCEOs) and other agricultural industry enterprises to boost agriculture industry development. The Association for snake-headed fish in YY town has been working on promoting “ecological breeding” for the “Black Beauty” brand by upgrading the breeding environment and wastewater treatment system. Since the issuing of the 2019 antimicrobial feed ban, the Association has started to invite an expert group to provide training about antibiotic-free breeding for farmers. At about the same time, the Association also coordinated in advancing drug use traceability technologies to encourage the “medicine-free” practices, whereby fishes are implanted with “T” shaped chips on the skin, which encoded information about the place of origin, farmers' names, and drug testing reports. Not all snake-headed fish farmers joined the Association and adopted the above practices, which according to an official of the ARA office of YY town is partly because the initiative is new, and partly because some farmers may not able to afford the costs of the investment (Interview 10, township agricultural official from D county).

The adoption of antibiotic-free feed and farming techniques are closely linked to top-down drug oversight enforcement and industry initiatives. Small-scale farms are left out of such initiatives because of a lack of resources and neglect of oversight mechanisms for ensuring food safety.

Case 2: The challenges of shifting to antibiotic-free feed

In XA town, a rabbit famer said he had started to use antibiotic-free feed for breeding rabbits since 2019. He and his family run a medium-scale farm breeding 5,000 cages of rabbits (1–7 rabbits per cage) and have been farming rabbits for nearly 20 years. He explained how he had shifted to antibiotic-free feed due to tightened regulatory oversight and felt this was unavoidable for larger farms like his.

“*The original complete formula feeds—because you want them to grow, and to grow quickly, the quantity of refined feed in the formula was high, and if drugs are not added—if antibiotics (kang sheng su) are not added—then the animals get diarrhea, their digestive tract can't take it. So because you both want them to grow quickly and to not have diarrhea, you have to add antibiotics (kang sheng su) or whatnot. That's how it used to be with rabbits. Now—I don't know about small farms, because we are a relatively large-scale operation, so we get the higher-ups coming to take samples, carry out tests, and do secret investigations a lot—one reason is for market safety, and the other reason is for our own peace of mind, so we started using antibiotic-free feed last year.”* (Interview 16, commercial farmer from D county)

This farmer admitted that the shift to antibiotic-free feed posed challenges for farm management as it means an increased risk of the rabbits getting sick. He mentioned adopting biosecurity measures to manage the situation while expressing view feeling that small farms lack the resources or capabilities for incurring the risks of economic loss in switching to antibiotic-free animal farming.

“*Previously, most of the farmers rearing animals placed treatment at the forefront—reating disease. Now we are approaching it the same as we do with people, preventing disease. So disinfection and disease spread prevention are things we have to keep up with. It used to be that we would wait until there was an illness before carrying out injections or giving the animals drugs. Now what we do has a regularity to it. Once a week, or in ordinary circumstances twice a month, we carry out disinfection. The drinking water needs to be sterilized, and the environment needs to be disinfected, to reduce the pathogens to as few as possible. In this way the occurrence of illness is also low. Adopting easy management like before can't keep up. If you were to use antibiotic-free feed with this then the risks would be comparatively great.”* (Interview 16, commercial farmer from D county)

“*It's a bit better for some of the smaller farms [in terms of the intensity of oversight], because they don't have so many people and resources……When there's nothing you can do—let's say you had a flock of chickens and there was an outbreak of disease. If you don't treat them then you'll have to cull the whole lot. If there's any medicine that can halt the disease then you'll definitely be going to the vet and asking an expert how to treat it. There's no other option. Because the rural people can't afford such losses. If you use fast and cheap products then there will be a secondary outbreak after a while. If you don't treat it properly, the losses will be too great. Then there would be way more drugs that would be used.”* (Interview 16, commercial farmer from D county)

#### 3.2.2. Smallholders' access to professional veterinary care and antibiotics

Smallholders have limited access to professional veterinary care. In China, there are two different veterinary systems, the Official Veterinarians System administrated by the government and the Licensed Veterinarians System administered by the Chinese Veterinary Medical Association. Licensed veterinarians are responsible for providing veterinary care services, while official veterinarians are responsible for law enforcement and control of animal diseases. The official veterinarians working in the vet station prioritized disease prevention tasks such as vaccination and animal epidemics prevention work. “*For humans, treatment is the priority, for animals, prevention is the priority.”* (Interview 31, township agricultural official from H district). Smallholders reported that it is too expensive to call a licensed veterinarian to come when their animals are sick. Although official veterinarians in the vet station provide some technical extension services, according to agricultural officials, the official veterinarian's team lacks capacity and human resources.

The interviewed commercial farmers reported a variety of sources to obtain veterinary care and extension services. Most commercial farmers purchase veterinary drugs directly from veterinary drug manufacturers. According to them, veterinarians employed by large drug manufactories can check their animals and offer them instructions on drug use. On-farm anatomy service can be provided when their aquatic animals are sick. The Farmers Cooperation Economic Organizations also provide veterinary services, farmers frequently use WeChat groups to seek veterinary guidance from technicians.

Smallholders mostly purchase veterinary drugs from local veterinary feed and drug stores. With limited access to professional care, smallholders turn to feed and drug store owners and peers for advice or use drugs based on prior experience of successful treatment of similar diseases.

Case 3: Use of human antibiotics for backyard animals

In YL town, H district, smallholders keep poultry (chickens, ducks, and geese) and sheep in their backyard, with numbers usually < 50, which are primarily for domestic consumption or offer to relatives or friends as gifts. There are also larger ones rearing over 200 birds that are sold for direct income. Both smallholders themselves and feed and drug store sellers reported that many smallholders routinely use human antibiotics bought from pharmacies for their backyard animals, sometimes for treatment of diarrhea and preventative purposes.

Smallholders can easily buy antibiotics in pharmacies without a prescription. “I just say I want several vials of penicillin and they let me buy it” (Interview 20, smallholder from H district). The feed and drug store owner in YL town reported it is common for smallholders to use their medical insurance card to buy human medicine for their animals.

“*The drugs sold in the pharmacies are actually from the rural medical insurance, but they aren't being used by people, but are being given to chickens. The monitors can't inspect this, they just say that it's the person who has the problem. ……The stuff they buy is cheap and ours is expensive here.”* (Interview 21, veterinary feed and drug store owner from H district)

When asked why they use human antibiotics, smallholders mentioned the successful treatment of animals using human antibiotics by both themselves and their peers. The efficacy of human antibiotics for animals is reinforced by word-of-mouth.

“*Sometimes the drugs bought from the vet pharmacy don't necessarily get the chickens better. If you see that a drug is working then you don't need to go to the vet pharmacy to buy anything. In any case, the drugs work.”* (Interview 20, smallholder from H district)

“*Q: How did your husband know to buy human penicillin to give to the chickens?*

*A: He heard people talk about it. Penicillin works well, you can go buy some. Knowing nothing about it, tried it out, and after giving them a little they got better right away.”* (Interview 20, smallholder from H district)

Participants reported human antibiotics to have the same or better effect than animal antibiotics. One smallholder expressed a lack of trust in the quality of veterinary drugs compared to those for human use, stating that he uses human gentamicin for sheep with diarrhea because human antibiotics are higher quality.

“*The human ones are more effective. The human ones are safer and more guaranteed. The ones for animal use could be fraudulent. Sometimes they don't work at all.”* (Interview 25, smallholder from H district)

In our study a smallholder also mentioned the reason he uses human penicillin is that penicillin is cheap and he considers it safer and as having lower “toxicity” than other antibiotics.

“*With these ones for prevention, using penicillin is best because it has low toxicity. I previously had a procedure for gall stones and they gave me cephalosporin. A relative of mine in the hospital told me not to use the cephalosporin and to use penicillin instead. Penicillin is inexpensive and good quality, and it's effective in treating disease.”* (Interview 19, smallholder from H district)

## 4. Discussion

This study reveals how the governance of antibiotic use in animal agriculture is unevenly implemented and antibiotic use practices vary among commercial farms and smallholders in eastern China. Drawing on interviews with various stakeholders in the agricultural sector, we show how antibiotic governance and use practices are conditioned by local resources and socio-economic position. Social sciences research on antimicrobial resistance has shown how antibiotic use in animal agriculture is driven by the socio-economic infrastructures rather than individual farmers' knowledge and practices (31–33). This study adds to the body of knowledge by providing further evidence on why antibiotic use practices differ between commercial and smallholders' farms in eastern China. In addition, it provides evidence to suggest that the division of bureaucratic oversight structures between agriculture (for production) and the market (for distribution and consumption) can make it difficult to ensure seamless governance across the whole food chain, while the bifurcation of veterinary registration and practice into parallel systems for disease control and clinical care may limit the availability of essential veterinary services.

Our study used qualitative methods and as such our sample size was limited. Our findings may not necessarily be representative of other agricultural settings in China. However, the results reported in this paper were consistent across the different groups of stakeholders who were independently interviewed and this provides confidence in their validity. The results were also consistent with existing studies on antibiotic use on farms in China that adopted other research methods (22, 23). As the interviews were performed in Mandarin, nuances of meaning may be lost in the translation process. We utilized professional academic translation services and then carefully examined the translations so that they represent interpretations of the participants' accounts as closely as possible.

The Chinese government has developed more stringent regulatory measures on veterinary drug use in recent years (34). Our study shows that despite progress made in reducing antimicrobial use in commercial farms through regulatory enforcement and inspections, smallholders were often left out of the oversight structure due to a lack of institutional and technological capacity of the agricultural administrations. The oversight work of the upper-level administrations focuses primarily on commercial farms, whereas the supervision capacity of grass-root level administrative teams is limited by a lack of human resources and technical support as well as a focus on disease prevention. Another reason contributing to this phenomenon is that smallholders are rarely considered as food safety supervision targets, due to the assumption since food animals kept by smallholders are only consumed within the individual household, drug use in these animals has minimal impact on the wider population. This view was expressed in interviews with both agricultural officials and smallholders.

Antimicrobial reduction action and antibiotic-free farming initiatives in this part of China mostly targeted commercial farms. The farms involved in these initiatives were trying to move from a treatment-based to a disease-prevention model, whereby farmers were lowering their reliance on antibiotics by using indigenous medicine alternatives and by introducing biosecurity measures. Such practices were promoted by training sessions and technical support that were closely tied to the value chain and depended on farmers' capacity to take on potential economic losses during the transition. These initiatives were not available to small-scale farms and smallholders, as they either do not have the money for financial investment needed to be involved in these antibiotic-free and biosecurity initiatives or were too vulnerable to bear any losses.

Compared with commercial farmers who can seek veterinary care and agricultural extension services from industry sources, small-scale farms and smallholders in China have limited access to professional veterinary services. China has undergone veterinary system reform since 2009 and has established a dual system of licensed veterinarians and official veterinarians. Nonetheless, our study reveals a dearth of qualified veterinarians available for farmers to seek clinical and technical services. Licensed veterinarians are expensive and official veterinarians are mainly responsible for disease prevention and control. Smallholders and small-scale farmers turn to informal providers like veterinary feed and drug store owners for instructions on drug administration or rely on peers and their own experiences. As reported in other LMICs, lack of access to professional veterinary care is likely to promote informal or unauthorized prescriber networks and becomes one of the drivers of antibiotic use (35).

Our study found that smallholders in H district in eastern China commonly use human antibiotics for treating their animals. The use of human antibiotics for animals has previously been reported in China (23) and other LMICs (36). Among the reasons for using human antibiotics in animals, the perceived better quality of human medicine and the dual use of human medicines for both humans and animals after purchasing are the main reasons for use of human antibiotics for pig farming in Yunnan, southwestern China (23). In their study of smallholders in Guatemala, Snively-Martinez (36) has discussed how human antibiotic use for poultry is driven by the absence of accessible and affordable veterinary medicines, and belief in the efficacy of human antibiotics. Similarly, a belief in equal or better quality of human antibiotics combined with easy over-the-counter access to antibiotics in pharmacies without prescriptions has contributed to smallholders' human antibiotic use in animals in eastern China.

Studies have shown that veterinary antibiotics can pollute the farm environment and pose health risks to humans (37). Considering the extensive links of AMR exposure under the One Health framework (5, 6, 38), efforts to integrate small-scale farms and smallholders in antibiotic governance are required to address the AMR burden systematically in China. Strategies to reduce antibiotic use and minimize the burden of AMR need to consider the political and economic conditions that drive inappropriate antibiotic use. In resource-poor settings, instead of prioritizing education campaigns to increase farmers' knowledge and awareness of AMR stewardship, more attention to addressing the infrastructure of veterinary regulation and provision of affordable and accessible veterinary care services is needed.

## Data availability statement

The data supporting the conclusions of this article will be made available through the University of Bristol data repository (data.bris) on an open access basis shortly after publication of this article. Requests to access this data should be directed to the authors who will provide a link without undue reservation.

## Ethics statement

The study was approved by the Institutional Review Board (IRB) of the School of Public Health, Fudan University. Before taking part, all participants received information about the study (verbally and in writing) and provided their written informed consent to participate in this study.

## Author contributions

WW, NW, CF, HL, and FY contributed to conception and design of the study and to acquisition of funding. WW, CM, ZD, and FY contributed to collection of data. BL, CM, ZD, WW, FY, and HL contributed to data analysis. BL wrote the first draft of the manuscript. All authors contributed to manuscript revision.
