# Supplementary material for: Antibiotic governance and use on commercial and smallholder farms in eastern China
Source: Front Vet Sci. 2023 Mar 17;10:1128707. doi: 10.3389/fvets.2023.1128707 (PMC10065158; doi:10.3389/fvets.2023.1128707)
Supplement: Supplementary file 1 [file Table_1.docx]

Supplementary Material

Antibiotic governance and use on commercial and smallholder farms in eastern China

Binjuan Liu^†^, Wei Wang^†^, Ziru Deng, Cong Ma, Na Wang, Chaowei Fu, Helen Lambert*, Fei Yan*

*** Correspondence:** Helen Lambert: [h.lambert@bristol.ac.uk](mailto:h.lambert@bristol.ac.uk) Fei Yan: fyan@shmu.edu.cn

# Supplementary Data

| Types of animal agricultural products | Detection indicators |
| --- | --- |
| Livestock products | clenbuterol(克罗特罗), ractopamine(莱克多巴胺), salbutamol(沙丁胺醇), chloramphenicol(氯霉素) |
| Poultry meat products | nitrofurans (硝基呋喃类)(including metabolites AOZ, AMOZ, AHD and SEM), chloramphenicol(氯霉素), ofloxacin(氧氟沙星), pefloxacin(培氟沙星), norfloxacin(诺氟沙星), lomefloxacin(洛美沙星), amantadine(金刚烷胺) |
| Poultry egg products | enrofloxacin(恩诺沙星), ciprofloxacin(环丙沙星), florfenicol(氟苯尼考) (including florfenicol amine氟苯尼考胺), ofloxacin(氧氟沙星), pefloxacin(培氟沙星), norfloxacin(诺氟沙星), lomefloxacin(洛美沙星), amantadine(金刚烷胺) |
| Aquaculture products | chloramphenicol(氯霉素), malachite green(孔雀石绿), nitrofurans(硝基呋喃类) (including metabolites AOZ, AMOZ, AHD and SEM), ofloxacin(氧氟沙星), pefloxacin(培氟沙星), norfloxacin(诺氟沙星), lomefloxacin(洛美沙星), diazepam(地西泮) |

**Supplementary Table 1.** Detection indicators for animal products for agricultural quality and safety supervision in China
